# The qualitative analysis of the nexus dynamics in the Pekalongan coastal area, Indonesia

**DOI:** 10.1038/s41598-022-15683-9

**Published:** 2022-07-06

**Authors:** Muhamad Khairulbahri

**Affiliations:** The Bureau of Research and Development, Jl. Pejanggik 1, Mataram, West Nusa Tenggara Indonesia

**Keywords:** Environmental impact, Urban ecology, Environmental sciences

## Abstract

Several studies investigated the dynamics of coastal areas, investigating some issues such as sea-level rise, floods, and water scarcity. Despite existing studies discussing coastal areas, there are limited studies investigating Asian coastal areas and their proposed solutions may not overcome extreme events. This study investigates the dynamics of the Pekalongan coastal area, Central Java, Indonesia. Despite efforts such as the development of dikes and groundwater pumping, people in Pekalongan have currently experienced more frequent floods and land subsidence that have led to larger inundated areas and people migration. Using the system archetypes, this study shows that the coastal area consists of renowned nexus elements (water, land, and food) and less recognized nexus elements (health and wellbeing). This means that changes in one nexus element may threaten other nexus elements, exacerbating problems in the observed system. For instance, unsustainable nexus actions such as overexploited groundwater tend to increase flooded areas, threatening people health, and inducing people migration. The system archetypes also show that the coastal area consists of Limits to Growth structures. As such, growth engines such as land-use change and groundwater pumping should be managed or restricted properly. Managing growth engines can prevent us from natural disasters such as floods and water scarcity. Likewise, as the system archetypes describe generic patterns and solutions, some findings of this study can be useful for the other coastal areas.

## Introduction

It is estimated that at least 250 million people live near the Asian low-elevation coastal zone (LECZ)^1^. Since, the LECZ is associated with tidal floods, flash floods, and land-use change, people who live in LECZ have already experienced negative impacts of natural hazards. As a large fraction of Asians lives in LECZ, Asian coastal cities have faced difficulties in providing comfortable living spaces for their inhabitants. It is due to population growth, economic activities, and climate change. Owing to their important roles in humankind’s daily life, urban areas, especially, coastal urban areas, expand more and more to support our life. As more employment and economic activities exist in the coastal areas, more people move towards the coastal areas^2^. More economic activities owing to urbanization lead to higher material consumption. As resources such as water and land are not abundant, coastal cities around the world have faced difficulties in providing a comfortable life for their inhabitants. This means that understanding the dynamics of LECZ is important as it enables us to balance resource supply and human consumption, leading to sustainable development.

Frequent natural hazards in coastal areas such as droughts, tidal floods, and flash floods have attracted some scholars to investigate the dynamics of coastal areas. Existing studies analyzed droughts or water scarcities^3,4^, sea-level rise^5,6^, and floods in Asian coastal cities^2,7^. In general, those studies proposed some solutions such as reused water^8,9^ and dikes or water tunnels for coping with tidal floods or flash floods^7,10^. However, many of the proposed solutions may not minimize natural disasters properly as seen in Pekalongan^11^ and Jakarta^7^. Besides, proposed solutions, especially dikes, are very expensive. So, development dikes are not widely applied in Asia.

Several studies^12–16^ pointed out that there are interlinked elements in coastal cities such as water, land, climate, and food. Unfortunately, existing studies that have investigated the nexus in Asian coastal cities are very limited^16^. In Indonesia, for example, most nexus studies were about the nexus in the Jatiluhur reservoir^17–19^. Besides, there are limited studies that have discussed important issues such as health and people migration. The latter is important as existing studies^20–22^ are concerned on the impacts of environmental changes and climate on health, wellbeing, and migration. Hence, this study aims to develop more sophisticated nexus connections in increasing the society awareness of the nexus dependence in the Indonesian coastal area. In other words, this study incorporates health, people migration, and other renowned nexus elements (water, energy, food, and land).

Among the Asian countries, Indonesia has the longest coastline^23^. Likewise, it is about 60% of the total Indonesians live in coastal areas. Besides, Indonesia has a coastline of about 81,000 km^24–26^, 14% of the world’s coastline^25^. Owing to these, this study is very important for Indonesia. The locus of this study is Pekalongan, an Indonesian coastal city, which is famous because of its *Batik* industry. As a rising coastal urban area, Pekalongan has experienced multiple unexpected incidences such as the variations in the groundwater table, insufficient water supply, and tidal floods.

Likewise, conducting research in the local coastal area is encouraged as existing studies^20,21,27^ confirm that the city governments such as the local government of Pekalongan have the responsibility to provide the healthy built environment and wellbeing for society. Hence, this study reinforces the responsibility of local (city) government to provide the healthy environment for local people.

This study starts with the first paragraphs, introducing some existing studies and their findings. Next, the research gap is described and results are described respectively. In the following sections, this study explains the implications of findings for other coastal areas before describing concluding remarks.

## Materials and methods

### Study area

**Figure 1:**
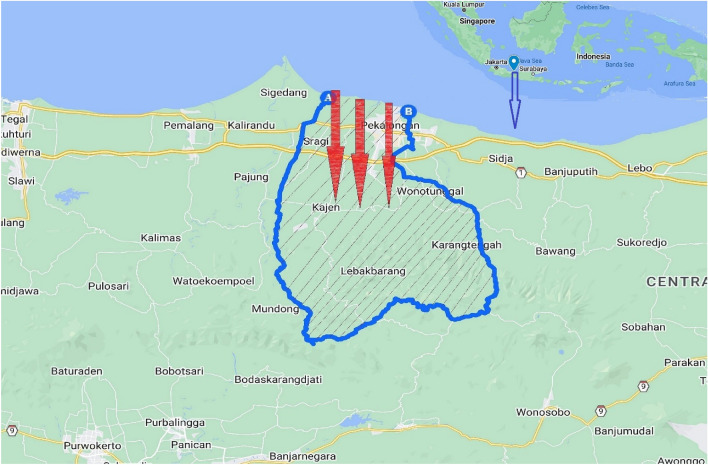
Pekalongan, Central Java, Indonesia^31^. The shaded area is Pekalongan and red lines show inundated areas after tidal floods.

Pekalongan is located in Central Java, Indonesia (Fig. 1). Specifically, Pekalongan is located between Semarang, the capital city of Central Java, and Cirebon, the main hub between Jakarta and surrounding areas. This city is famous because of its *Batik*, an Indonesian traditional dress, and the Indonesian national costume. Owing to its popularity, *Batik* has been transformed into different modern product variations and it is categorized as one of the UNESCO heritages^28^. Due to its high *Batik* quality, Pekalongan is awarded as the World *Batik* city^29^.

Geographically, Pekalongan is between 6 50′ 42″–6 55′ 44″ South Latitude and 109 37′ 55″–109 42′ 19″ East Longitude^30^. Its elevation is about 1 m above sea level and its area is about 4525 ha, dominated by urban housing and farming areas^30^. Most livelihoods are fishermen and farmers as its land is close to the Java sea and the second most land is farming areas^30^.

**Figure 2:**
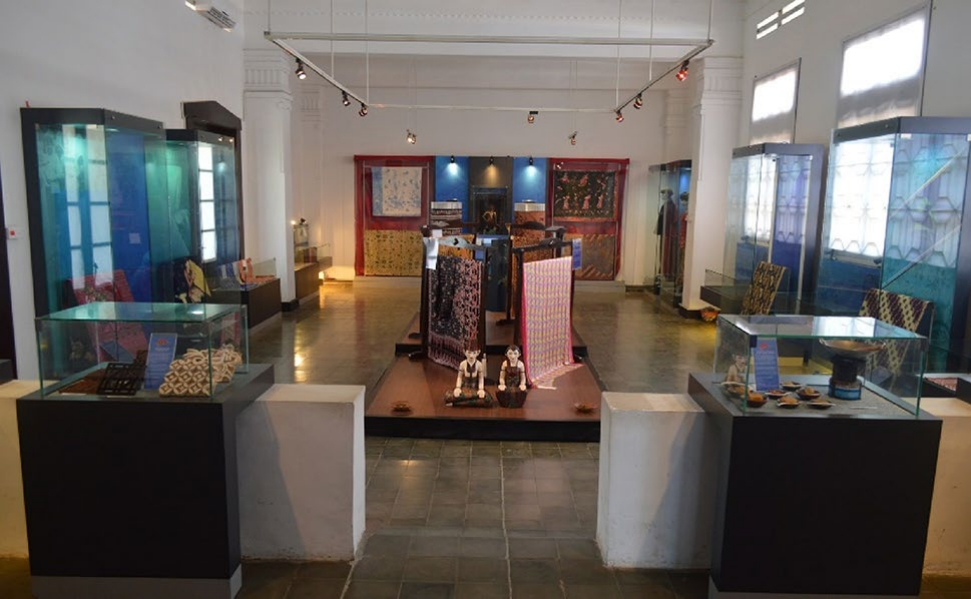
Batik collections in the Batik Museum Pekalongan^33^.

Pekalongan’s population is about 1,200,000 people and its population has increased by about 2% a year^30^. In the last 4 years (2016–2019), Pekalongan’s economic growth has been about 3% year. Due to its heritage value and its economic importance, the government has constructed the *Batik* museum to conserve *Batik* products (as seen in Fig. 2). All of these efforts have put Pekalongan as the largest creative industry in Java^32^.

### The system archetypes

The system dynamics (SD) approach has been applied in several different fields such as agriculture^34^, and public health^35^. The SD approach relies on understanding the system structure. Once people can capture the system structures, they can explain and predict the system behavior, the system performance, and the system failures. As consequence, after understanding the system structure, scientists can propose solutions to overcome bottlenecks in the observed systems^36^.

The system dynamics also concern interdependency and the complexity of studied sectors. The interdependency and complexity are corresponding with dynamic feedback within studied sectors. Through dynamic feedback, the effects of one sector to other sectors and vice versa are examined and quantified^37^. Investigating the dynamic feedback and uncertainty leads to minimizing possible unintended consequences. Preventing unintended consequences is important as existing studies explain that proposed solutions lead to possible unintended consequences such as the fish outbreaks^17^.

The SD approach has two different tools: the system dynamics models and the system archetypes. While the SD models can quantitatively explain the system structure and behavior, the system archetypes explain the observed system qualitatively. In this study, the system archetype is applied for three main reasons. The first reason is the system archetypes are the universal patterns that exist in different kinds of the observed systems. The second reason is the system archetypes enable us to find bottlenecks and generic solutions to increase and sustain the system performance. The third reason is the coastal areas are the complex system^38^ and the system archetypes are a suitable tool for capturing connections, dependence, and feedbacks in the complex systems^34^.

Likewise, the system archetypes, a qualitative tool of the system dynamics, have been applied in varied nexus studies such as the Jatiluhur reservoir^17^; the European organic food^39^; and the natural resource management^40^. This means that the system archetypes are the applicable and reliable tool for nexus studies. Some useful strong points of the system archetypes are that system archetypes do not need abundant data and complex mathematical relationships. Narratives, for example, can be used to compose the system archetypes structures. Although system archetypes can be composed of narratives, system archetypes can provide insights into observed studies, so we can predict possible problems and define possible solutions^41^.

**Figure 3:**
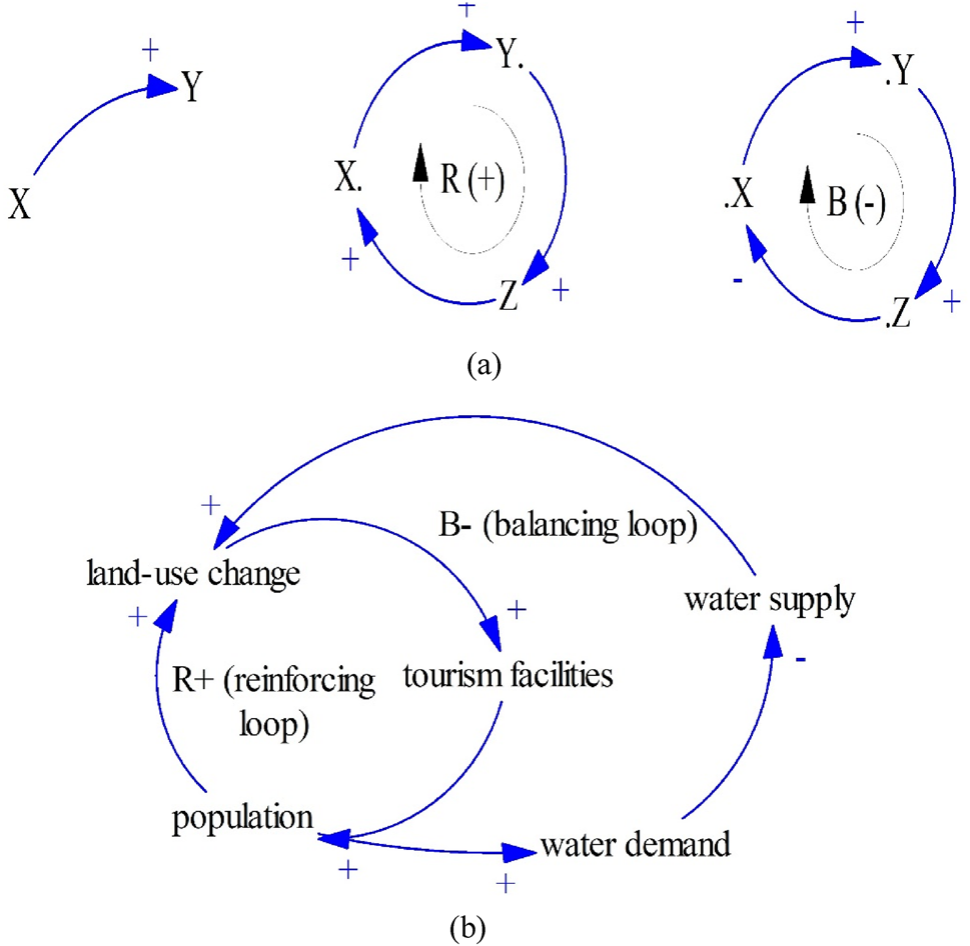
A causal link and feedback loops (**a**), and the Limits to Growth archetype (**b**).

To date, there are a lot of identified system archetypes structures such as The Limits to Growth, Shifting the Burden, Eroding Goals, and Escalation. A generic structure of the system archetypes consists of a combination of two or more feedback loops including balancing and reinforcing loops. A feedback loop is a combination of two or causal links between elements that are connected in such a way, one eventually returns to the first element. For example, if a change in variable A directly causes a change in variable B which directly causes a change in variable C, which in turn directly causes a change of our initial variable A, then we are dealing with a feedback loop (see Fig. 3a).

To identify the system archetypes, there are three consecutive steps. We collect relevant literature review and in the second step, we compose narratives based on existing studies. Based on narratives, data, and literature review, we describe identified system archetypes and explain generic solutions for identified system archetypes^42^.

Among identified system archetypes, perhaps, the Limits to Growth structure is the most frequently identified structure. As seen in Fig. 3b, a combination of a reinforcing loop (R) and a balancing loop (B) is called The Limits to Growth^42,43^.

Figure 3b reproduces a usual dynamic pattern found in the urban areas. A reinforcing loop (R = land-use change–tourism facilities–population) tells us that population leads to more land-use change. And land-use change leads to more tourism facilities such as hotels and restaurants. The growth of urban areas is bounded by a limiting factor, water supply. A balancing loop (B = population–water demand–water supply) tells us that water as a limiting factor cannot support the growth of urban areas as water demand will deplete water supply, leading to restricted land-use change. This case is found, for instance, in Benidorm, Spain^44^.

### Research approach

The coastal system is complex as it consists of sophisticated interactions between human beings and natural disasters. Natural disasters are, for instance, tidal floods, storm surges, and sea-level rise. Natural disasters may threaten assets or capital in coastal areas.

Since the coastal area consists of complex interactions, a combination of data and a literature review was collected during this study. Based on collected data, we can identify system archetypes, proposing some generic solutions^42^. In case data is insufficient, narratives and/or storylines can be summarized to generate meaningful system archetypes^34,43^.

**Figure 4:**
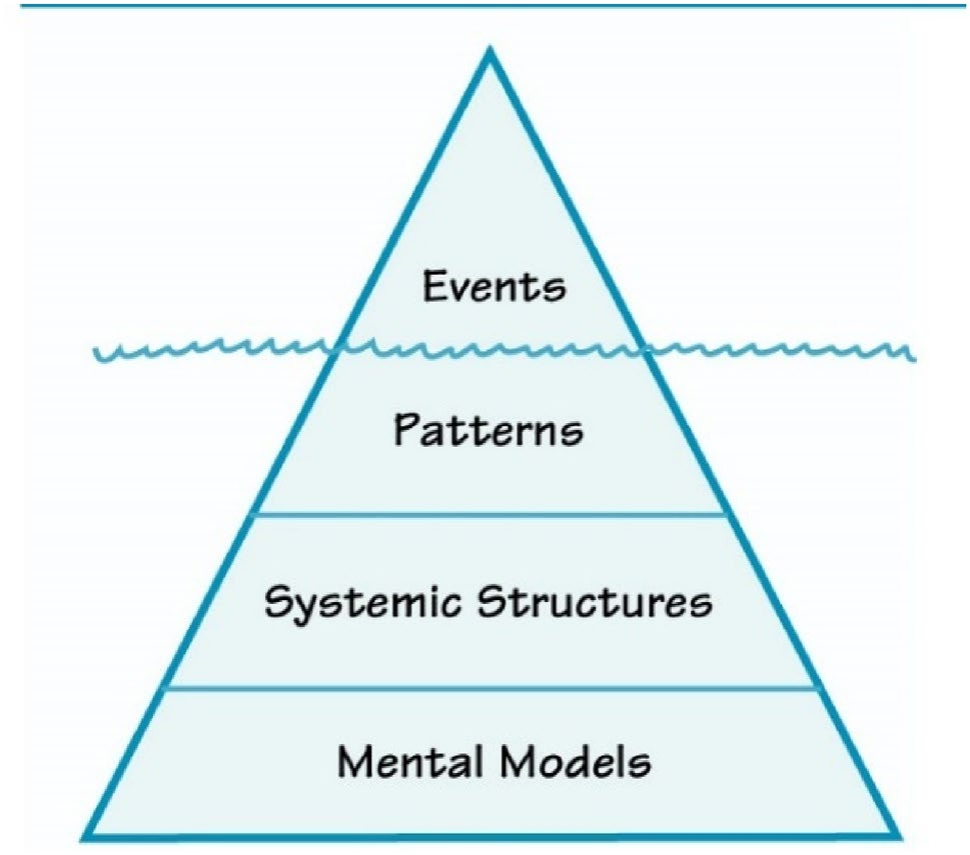
Four levels of system thinking^41^.

To convert collected data and narratives into the system archetypes, this study conducts four levels of system thinking steps^41^ as seen in Fig. 4. Those four levels are events, patterns, systemic structures, and mental models. Each level is explained in the following paragraphs.

At the event level, key issues of the observed systems are described. Besides, decision-makers usually focus on observed events and then make decisions on a series of observed events. As observed events may camouflage causalities of observed systems, decisions based on observed events may solve symptoms but they usually do not solve real problems^41^.

There are about 1100 *Batik* companies in Pekalongan and about 81% of them are small to medium *Batik* enterprises^32^. Of Gross Domestic Product (GDP), Pekalongan’s manufacturing sector contributed to about 23% of Pekalongan’s GDP^45^. It is estimated that about 70% of the manufacturing sector is supported by the *Batik* industry^46^. The *Batik* industry also contributed to about 50–80% of the total employment in Pekalongan^46^. The increasing roles of the *Batik* industry have led to an increasing water demand^30^.

At this level, decision-makers took a critical decision based on an important event, an increasing water demand. That is, allowing the *Batik* companies to extract groundwater. Extracting groundwater is not a real solution to an increasing water demand as an excessive groundwater extraction led to land subsidence and frequent tidal floods^47,48^.

In the second level, the real-world patterns can be collected through behavior over time graphs^43^. In general, behavior over time (BOT) graphs show how observed systems act or change over time. As the second level is based on data, it tends to provide more meaningful information.

**Figure 5:**
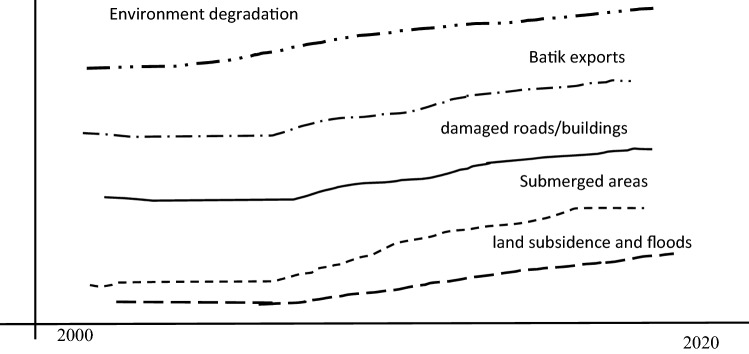
BOT graph of key variables in Pekalongan.

Figure 5 illustrates the BOT graph of some key variables in the Pekalongan coastal area over recent years. The main aim of the BOT graph is to provide readers information about directions of the key variables in the observed systems^41^. Owing to this, the vertical axis and horizontal axis of the BOT graph show performance measures of interest and time consecutively^41^. Thus, the BOT graph is drawn in a rough sense without exact values attached^41^.

Although the BOT graph or Fig. 5 does not display exact values, the dynamics of key variables are well verified by relevant supporting evidence. For instance, the Pekalongan coastal area has experienced a gradual increase in its Batik export from 3 billion Indonesian Rupiahs in 2000s to 17 billion Indonesian Rupiahs (IDR) in 2020^30,49,50^. However, at the same time, Pekalongan has experienced environmental degradation^47,51,52^. For instance, due to overexploited groundwater, Pekalongan has experienced an increasing land subsidence about 7.7–10.5 cm/year^53^ in 2009 to about 10–14 cm/year^54^ in 2015, leading to larger submerged areas over time.

Owing to land subsidence, in the early 2000s, it was estimated that about 9% of the total Pekalongan area was submerged after floods and by 2020, about 29% of the Pekalongan coastal area was submerged^55–58^, leading to larger damaged roads and houses^59,60^. Next, land subsidence has also led to more frequent floods in Pekalongan, from 0–1 floods/year to 2–5 floods/year^61^. This means that a prosperous Batik export is negated by environmental degradation^46^.

In the third level, the systemic structure, connected multiple actors interact and affect each other. Existing causalities and interactions between multiple actors can be summarized in a causal loop diagram^37,41^. A simplified causal loop diagram (Fig. 16) will be explained after identified system archetypes are explained.

Through the mental models, the fourth level, we can analyze observed systems and correct our mental models that are based on our beliefs, values, and assumptions^41^. Next, based on narratives and collected data, it is found that integrated planning is required to achieve sustainable development in Pekalongan. It also found that man-made acts such as groundwater exploration and land-use change lead to environmental degradation. Thus, this study proposed the nexus approach in analyzing the dynamics of the coastal area in Pekalongan, Indonesia. The nexus approach is further explained in the section “The nexus between water, energy, food, and land (plus health)”.

#### The nexus between water, energy, food, and land (plus health)

One promising approach of the integrated natural resource management is nexus. In general, nexus considers that connected elements are dependent on each other. This means that it is not possible to discuss one element since each element is dynamically dependent on other elements^62^. In Pekalongan, once water or groundwater is explored excessively, people have experienced land subsidence^47^. Another example is more land-use change leads to climate change that causes a sea-level rise or inundated areas^47^. This means that nexus enables us to see connections between water (groundwater), land (land subsidence and inundated areas), and climate change. It is thus this study that applies the nexus concept to analyze interactions among multiple actors in Pekalongan. The system archetypes are applied to visually identify interactions and obtain generic solutions to critical issues in Pekalongan, Central Java, Indonesia.

The role of the system dynamics approach in the nexus approach can be detected in the 1970s after a monumental publication of The Limits to Growth^63^. Another study^63^ reminded us of possible imbalances between the world population and food production owing to resource depletion and industrialization. That study also encouraged us to support sustainability in water, food, and energy for humankind’s existence^62^. In short, the nexus approach is in line with the application of the system dynamics application in the nexus studies.

Moreover, there is an increasing interest among academia in the nexus between water, energy, food, and land^62,64–66^. This growing interest is due to important premises. The first premise is the nexus context requires analyzing different elements of nexus simultaneously as each nexus element dependents the each other. The third premise is an imbalance between resource availability and high resource consumption requires the nexus management to manage resource demand and resource supply.

The nexus means a connection of a couple of elements that interact and affect each other. As each element affects other element(s), discussing one nexus element will lead to unsustainable other nexus elements. This may lead the community to resource scarcity. For instance, in the nexus between water, food, land, and energy, researchers who focused only on fish production can sacrifice energy supply and/or water quality^17^. It is thus when people discuss nexus studies, people must consider the importance of the connections between nexus elements and how a nexus element determines other nexus elements and vice versa.

**Figure 6:**
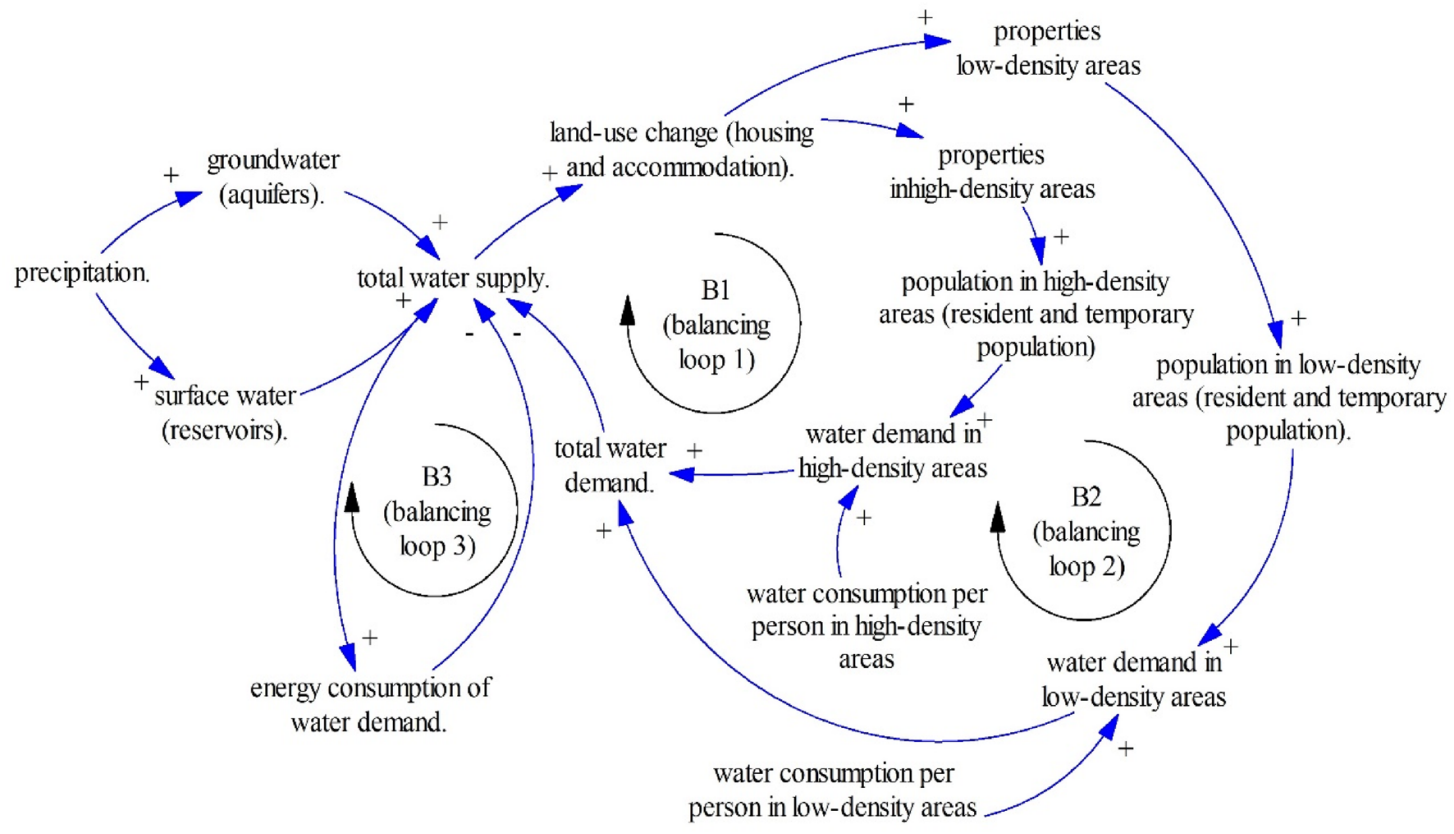
A simplified node of nexus elements in the Marina Baixa County^44^.

A possible nexus connection based on the system archetype approach can be seen in Fig. 6. This figure is a simplified summary of the nexus between water, energy, and land in the Marina Baixa^44^. Water supports the population in low and high-density areas, while water extraction increases energy consumption in the Marina Baixa. The Marina Baixa is a drought area in Alicante, Spain that has experienced droughts in the last decades. Droughts have happened as Benidorm (one municipality in the Marina Baixa) has attracted a million tourists every year, leading to high water demand in this drought area. Once people focused on surface water and groundwater, there is increasing energy consumption. Moreover, as people convert land to build hotels and residential areas (land-use change increases due to urbanization and tourism), water supply increases (droughts occur). This means when we only focus on one nexus element (either water or land), we may sacrifice other resource availability. This example should be recognized as the previously mentioned premise that nexus elements must be determined simultaneously. Otherwise, we will sacrifice or decrease other resource availability once we focus only on one resource or one nexus element.

The nexus study as seen in Fig. 6 shows that the system archetype approach can be applied to investigate the nexus studies. Moreover, this study takes one step ahead to include health, and wellbeing in the nexus studies. In other words, as climate change and environmental changes may threaten our health and wellbeing^21^, this study combines the renowned nexus elements (water, land, and food) with health and wellbeing.

## Results and discussion

### Land-use change, consumption, and climate change

Pekalongan has relied on agricultural areas and the flourishing *Batik* industry. Owing to industrial development and housing development, Pekalongan has experienced land-use change. Land-use change has happened as people migrate to Pekalongan city to support industrial development, especially *Batik* industries.

There are about 2000 *Batik* companies in Pekalongan and about 81% of them are small to medium *Batik* enterprises^32^. As small and medium enterprises are labor-intensive industries, the Batik industries contribute to about 50–65% of the total employment in Pekalongan^46^. *Batik* industries have increased accumulated capital such as factories, and machinery. Total capital in the Batik industry has increased from IDR 72 billion^67^ in 2000s to IDR 450 billion in 2020^30^.

**Figure 7:**
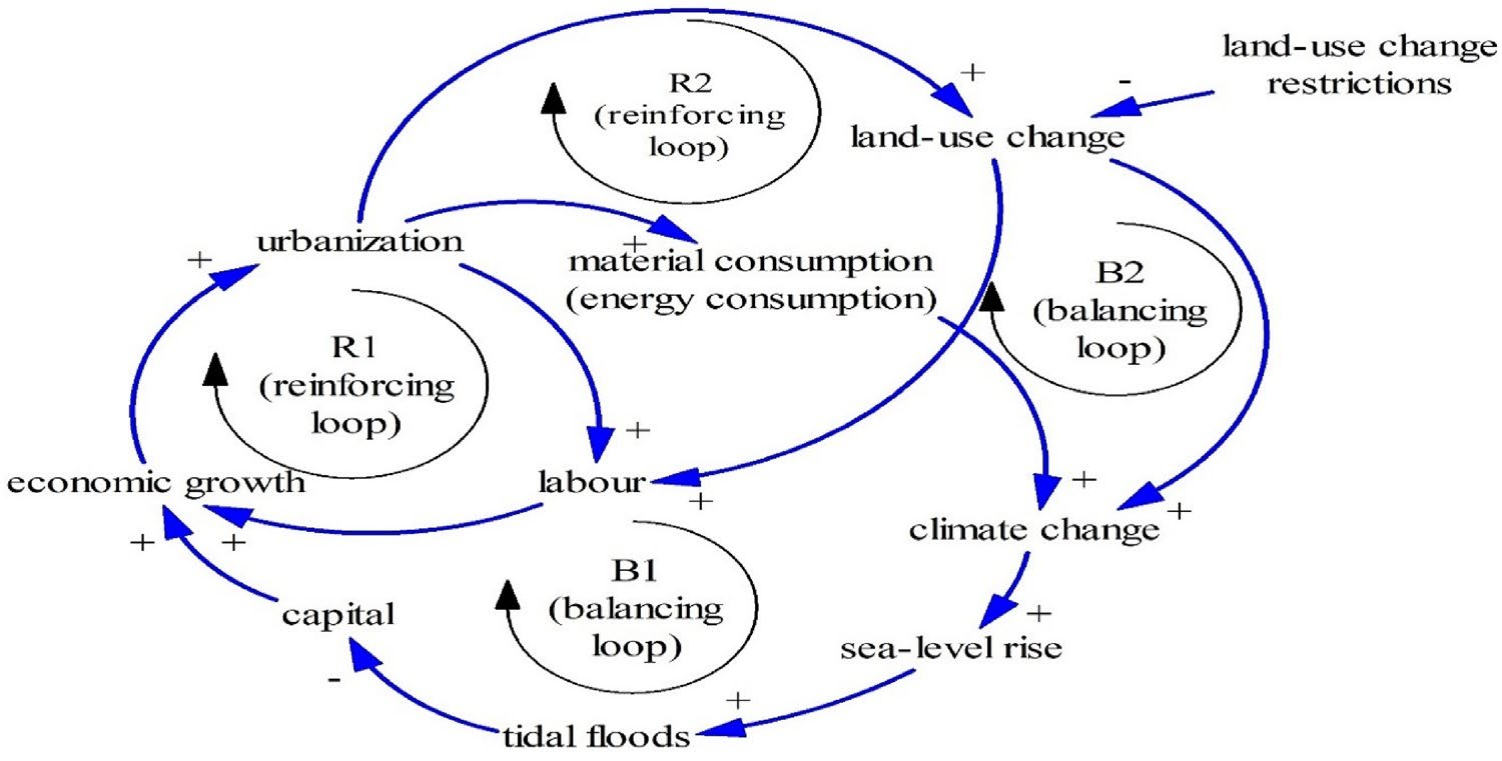
The Limits to Growth #1 (A notation of “#1” is used for numbering the Limits to Growth).

Interactions between capital, economy, and land-use change in Pekalongan and its surrounding areas can be represented in the Limits to Growth structure (Fig. 7). The growth engine of this interaction is economic growth supported by two reinforcing loops: R1 (economic growth–urbanization–labor) and R2 (economic growth–urbanization–land-use change–labor). In this case, land-use change and urbanization can increase capital and workers. It is acknowledged that capital and labour are two important factors of the economic development^68^.

**Figures 8:**
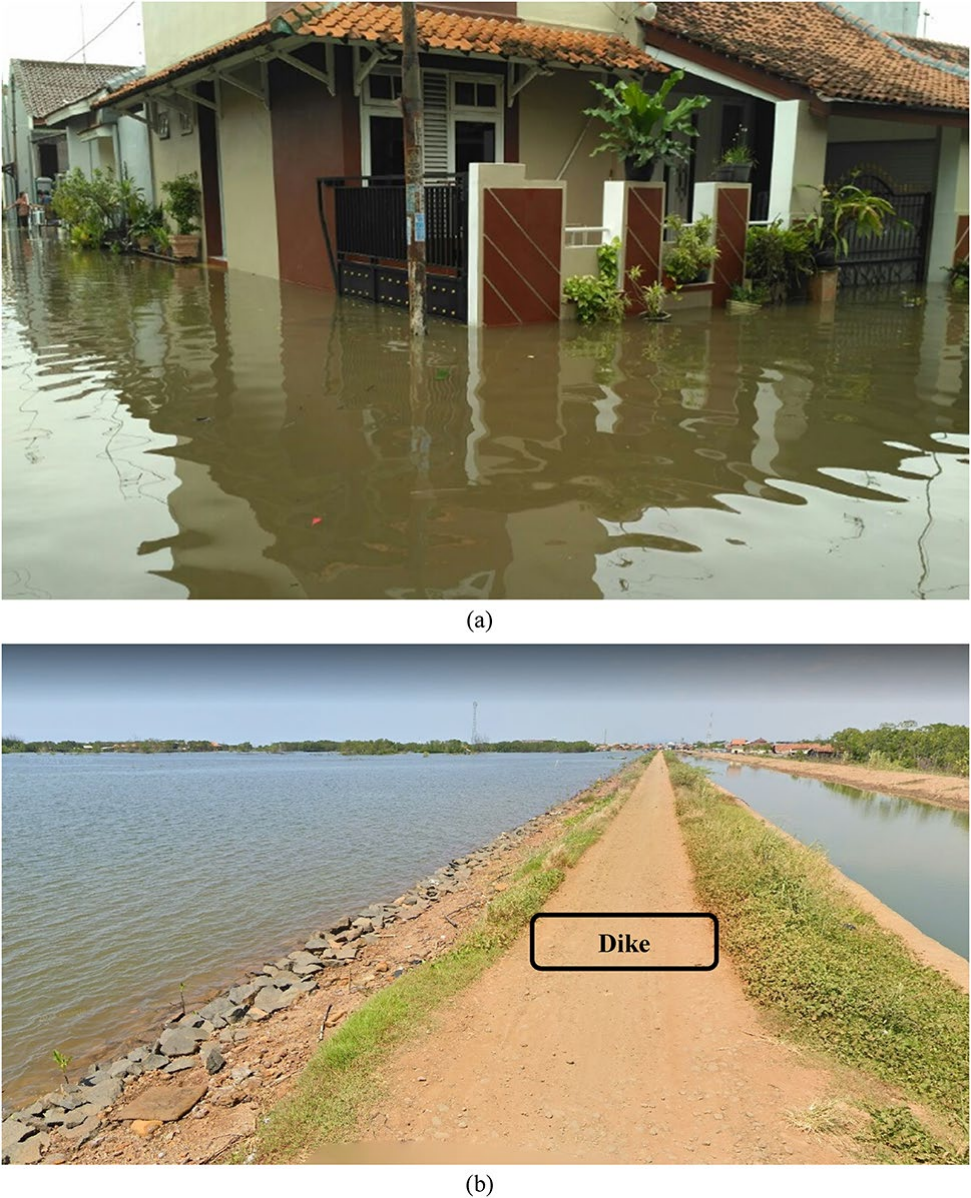
Flooded houses after tidal floods (**a**)^72^. A dike in the Pekalongan coastline (**b**)^73^.

Urbanization, on the one hand, leads to more material and energy consumption which induces climate change through increasing greenhouse gases after material and energy consumption^69,70^. Increasing material and energy consumption after urbanization is also noted by the local statistics bureau of Pekalongan^30^. Two balancing loops, B1 and B2, prove that climate change may threaten economic growth as B1 and B2 induce climate change through higher material consumption (economic growth–urbanization–material consumption–climate change–sea-level rise–tidal floods–capital) and land-use change (economic growth–urbanization–land-use change–climate change–sea-level rise–tidal floods–capital). Figure 7 also shows that tidal floods owing to sea-level rise associated with climate change can damage capitals^47,71^ such as offices, and houses alongside the Pekalongan coastline as seen in Fig. 8a.

Possible solutions to escape from this situation are restricting urbanization or land-use change as it induces higher material consumption and land-use change, two causes of climate change.

**Figure 9:**
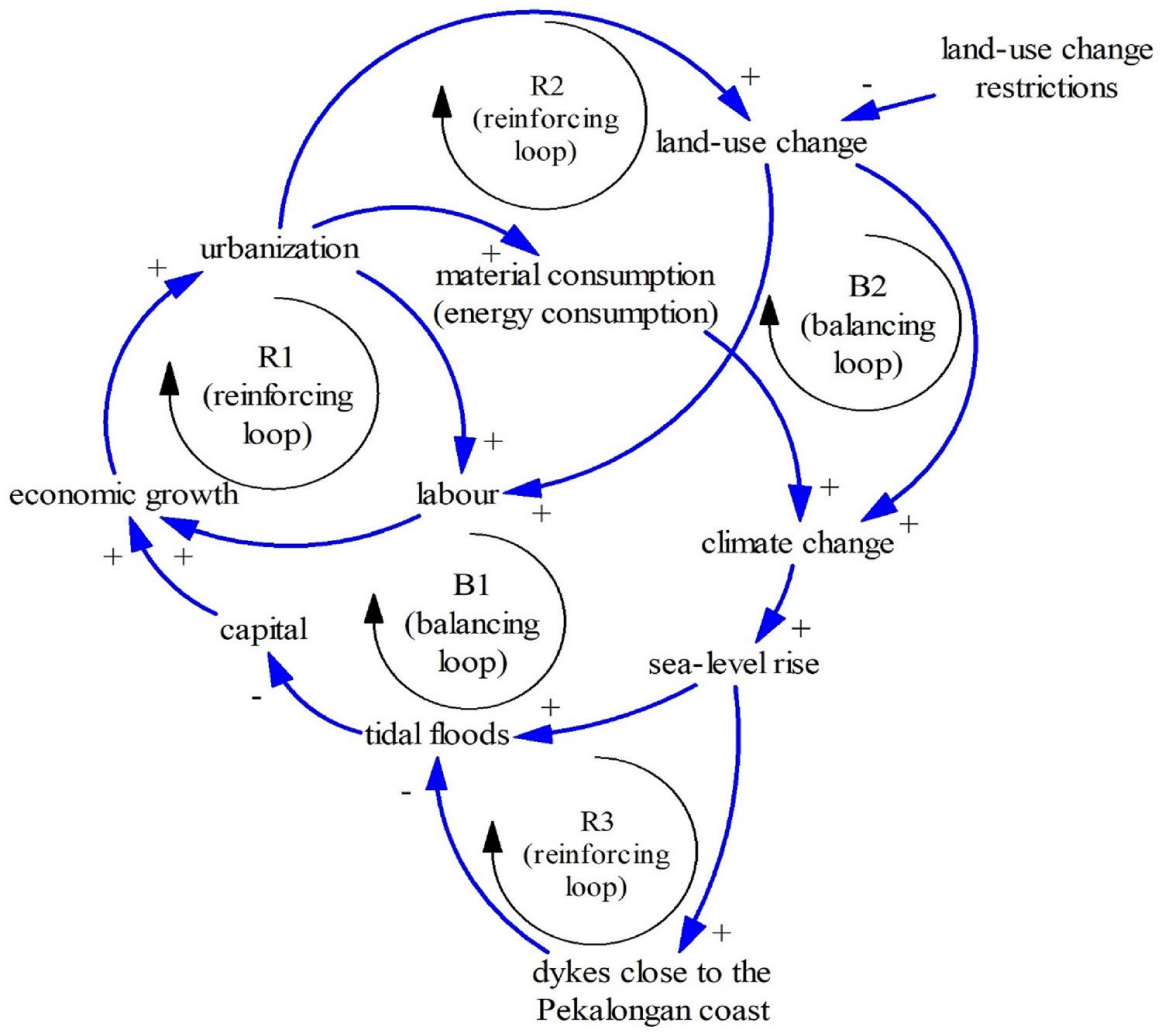
The Limits to Growth #2.

To minimize tidal floods, the government has built a dike (Fig. 8b). This also can be drawn in Fig. 9 as a reinforcing loop (R3 = economic growth–urbanization–material consumption–climate change–sea-level rise–dikes–tidal floods–capital)^51,74^. This reinforcing loop (R3) means that massive urbanization or land-use change leads to sea-level rise and tidal floods. Without managing or hindering land-use change, tidal floods may not be controlled properly.

### Water and food

Land-use change has two possible effects: more runoff^75^ and rising water demand^69,76^. Land-use change, in general, diminishes public green areas or forests that function as a source of infiltrated water. Less infiltrated water means more runoff, threatening the water supply. Furthermore, land-use change can increase water demand, leading to water deficits.

In Pekalongan, the government allowed people and companies such as *Batik* factories to discharge local aquifers owing to rising water demand. Please note that groundwater discharges need a water pump (located close to resident housing) while commercial water companies apply some capital such as water pumping stations, long pipelines, and water pumps. In other words, discharged groundwater is cheaper than commercial water. Thus, it is not surprising that most people consume groundwater than water from commercial companies.

**Figure 10:**
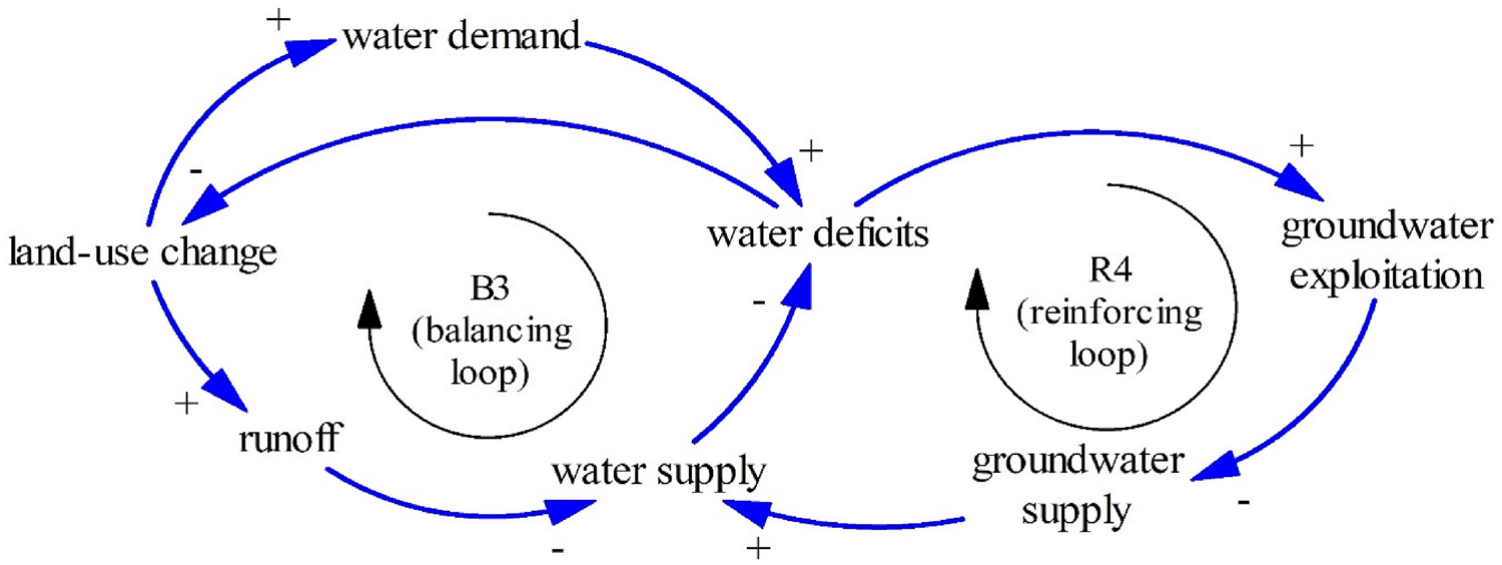
The Limits to Growth #3.

Dynamic relationships between land-use change, water demand, and groundwater lead to the Limits to Growth structure (R4–B3) as seen in Fig. 10. The Limits to Growth structure (R4–B3) reminds us to control land-use change, sustaining green spaces that absorb rainfall to increase water supply and minimize runoff.

Owing to massive groundwater exploitation, Pekalongan has experienced land subsidence^47^. The land subsidence leads to decreasing surface land, leading to more incoming seawater due to sea-level rise. Several studies^47,77^ stated that the height of incoming seawater was about 50–100 cm or higher and it has inundated a large part of Pekalongan and its surrounding areas in the last decade^47,77^. Moreover, existing studies^47,77^ reported that tidal floods may last several hours^60^, damaging housing, public facilities, and business properties. It was estimated that the economic loss due to tidal floods is projected at about IDR 7 trillion (about USD 50 billion)^71^. The local governments also reported an increase in the number of affected people after floods from 55 people in 2010s to over 100,000 people in 2020^61^. The number of affected people has increased owing to increasing affected areas from 9 to 29% of the total Pekalongan areas^55–58^.

**Figure 11:**
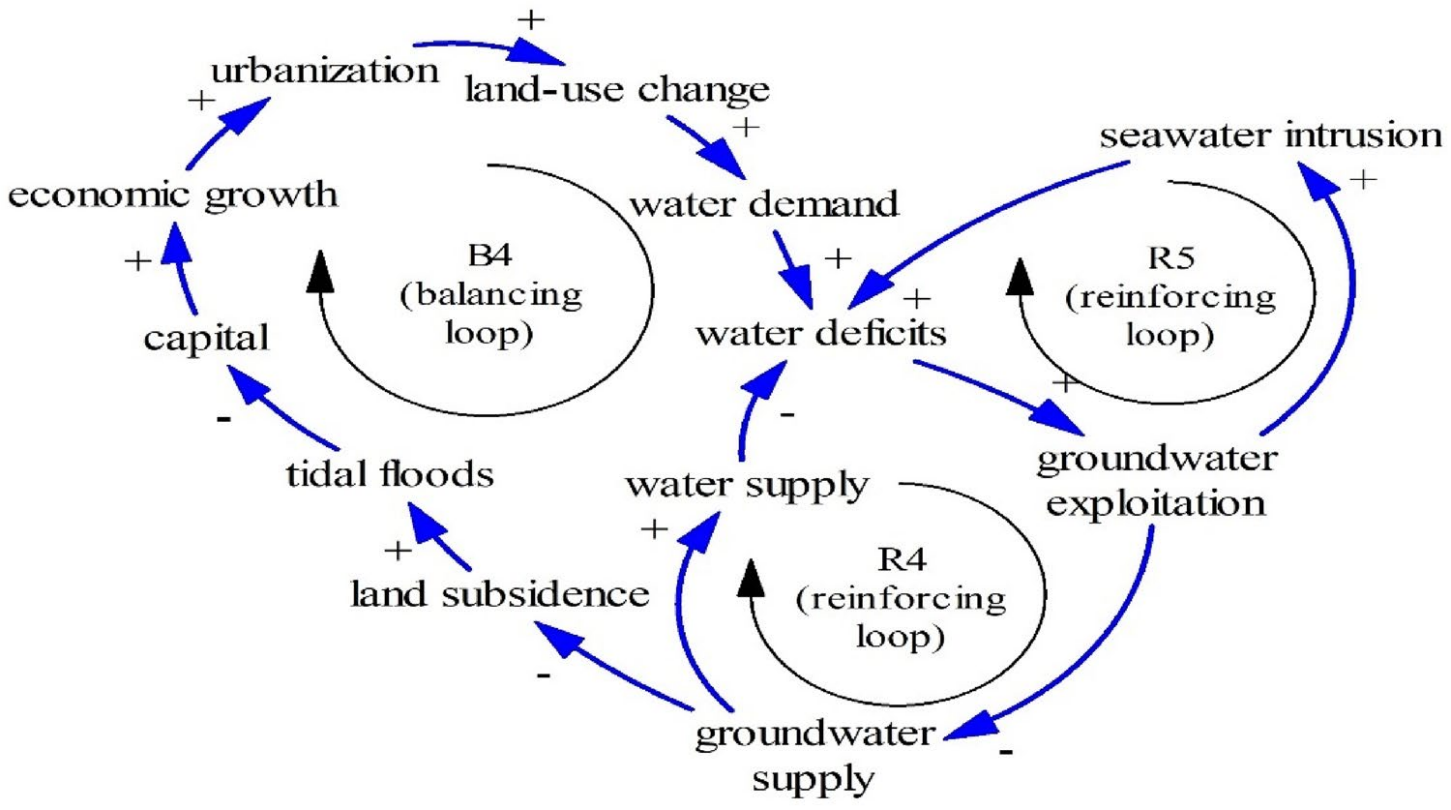
The Limits to Growth #4.

**Figure 12:**
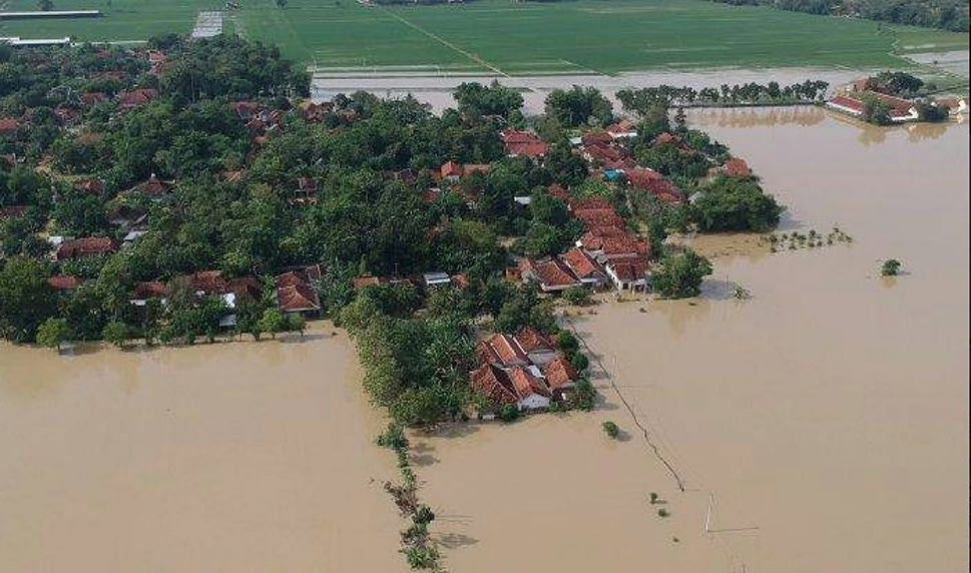
Inundated areas after the dike construction^78^.

As seen in Fig. 11, two reinforcing loops (R4–R5) and one balancing loop (B4) are encapsulated as the limits to growth structure. Groundwater has many functions such as blocking seawater intrusion and impeding land subsidence. Once people excessively exploit groundwater (R4), people are likely to experience seawater intrusion as R5 (groundwater exploitation–seawater intrusion–water deficits). Owing to land subsidence and despite the development of dikes, people in Pekalongan have still experienced tidal floods (Fig. 12).

**Figure 13:**
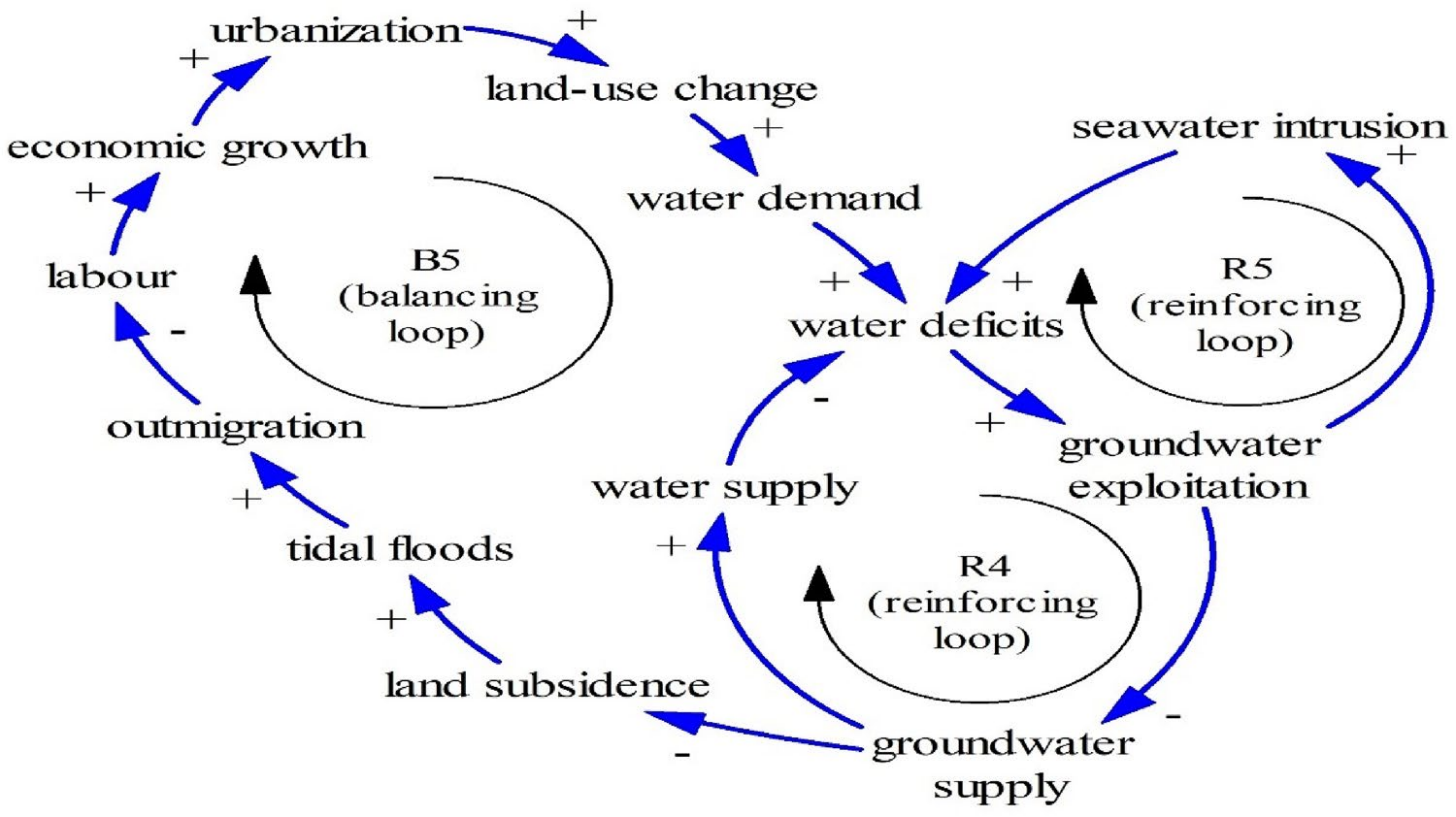
The Limits to Growth #5.

**Figure 14:**
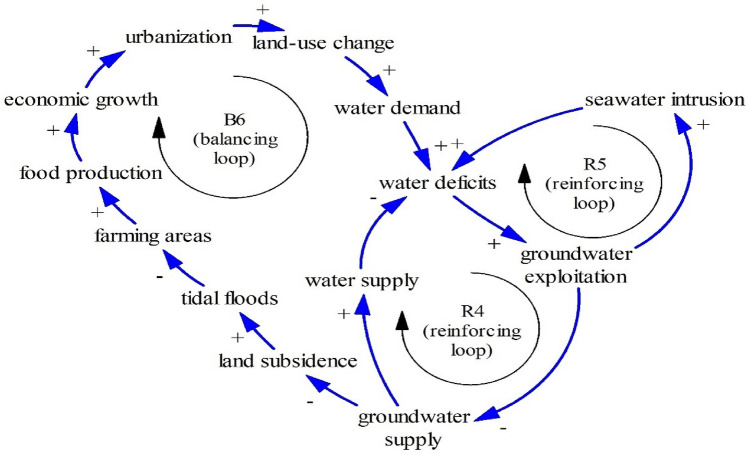
The Limits to Growth #6.

Figures 13 and 14 show other Limits to Growth structures. While Fig. 9 shows that tidal floods may damage capital, Figs. 13 and 14 show that tidal floods cause outmigration (B5) and less food production (B6) respectively. As people need a comfortable life, inundated houses and inundated properties after tidal floods induce outmigration^1, 79^. Moreover, due to people relocation, coastal areas experience labor shortages, threatening economic activities as seen in B5.

Tidal floods may increase soil salinity, leading to infertile farming land and decreasing agricultural production^47,71^. As farming areas decrease, food availability declines (B6). This means that exploited groundwater and sea-level rise associated with climate change threatens interlinked nexus elements including land (farming areas), water (groundwater), food (farming areas), and economy (damaged capital and lack of labor).

### Health and wellbeing

**Figure 15:**
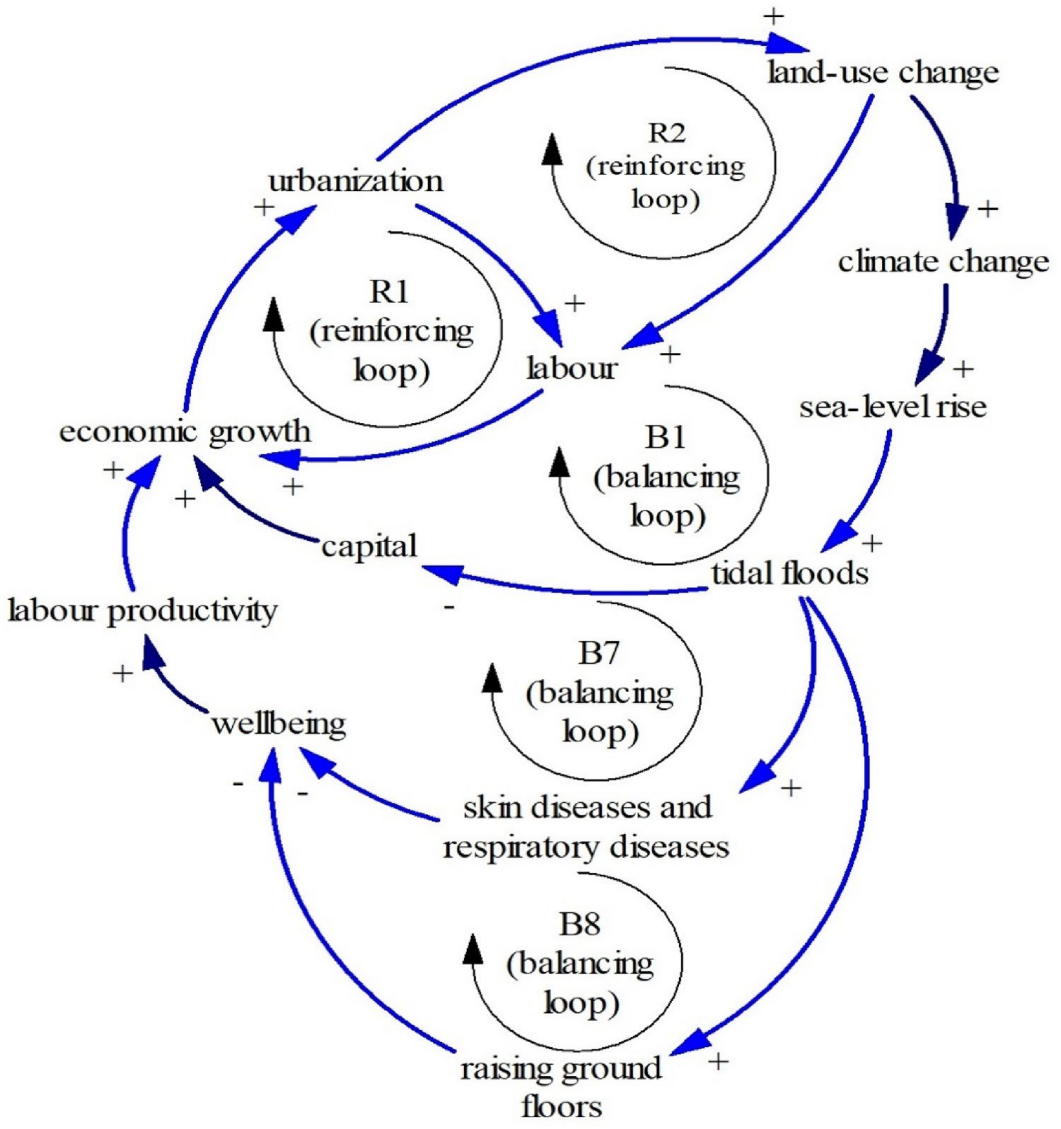
The Limits to Growth #7.

Tidal floods in Pekalongan have threatened society health^11,59^ and wellbeing^80^. As previously mentioned, owing to inundated areas, some green and public spaces in Pekalongan are gradually decreased. Tidal floods also tend to threaten people’s health as tidal floods induce bacteria that lead to dermatitis or other skin diseases, and respiratory tract infections^11,59^. Another impact of the tidal floods is structural adaptations such as raising ground floor^71^, leading to limited living spaces. Limited public spaces and limited living spaces can decrease society’s well-being^80^. Figure 15 shows a simplified connection between tidal floods, health, and wellbeing.

Two balancing loops (B7 = economic growth–urbanization–material consumption–climate change–sea-level rise–tidal floods–skin disease–wellbeing–labor productivity) and (B8 = economic growth–urbanization–material consumption–climate change–sea-level rise–tidal floods–raising ground floors–wellbeing–labor productivity) show that less wellbeing and less healthy leads to less labor productivity^81^. As seen in Fig. 15, tidal floods increase the number of people with given diseases, showing an additional nexus element, health. Likewise, tidal floods cause people to raise their ground floors so their houses are not flooded, lessening society’s wellbeing. Hence, tidal floods through wellbeing and health may influence economic growth through labor productivity.

### A simplified causal loop diagram

**Figure 16:**
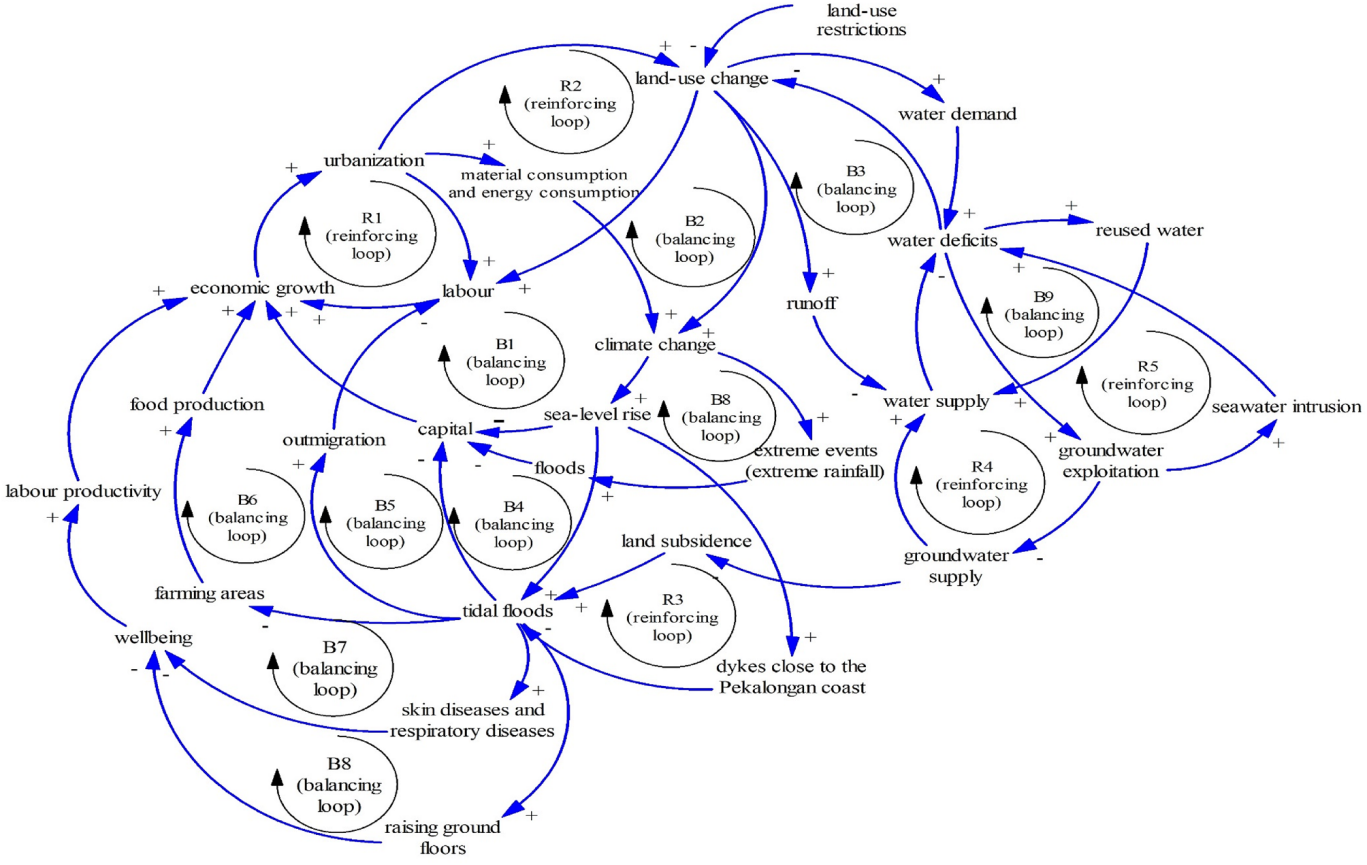
A causal loop diagram.

**Figure 17:**
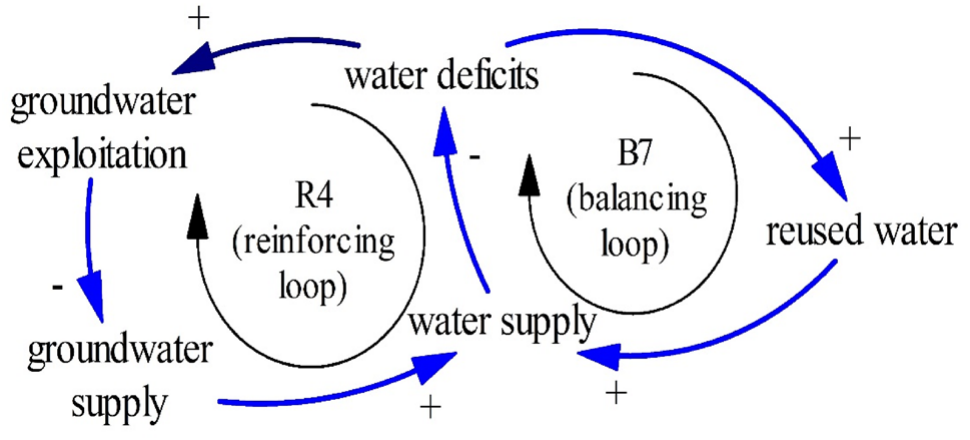
The Limits to Growth #8.

The combination of previously mentioned structures can be summarized in Fig. 16 as a simplified causal loop diagram (CLD). The CLD is dominated by the Limits to Growth structures. There are 6 structures of The Limits to Growth in total (the last of the Limits to Growth is explained in the following paragraphs). This means that the development of coastal areas, in general, is hindered by existing barriers such as water availability and sea-level rise. As the Limits to Growth structure may be remedied by removing barriers^41^. Owing to this, reused water should be one sustainable solution (Fig. 17).

One possible solution is reused water as seen in Fig. 17. Reused water can increase water availability so people can escape from overexploited groundwater, land subsidence, and abandoned farming land. Figure 17 shows another structure of the limits to growth (R4–B7). This structure inhibits overexploited groundwater associated with land subsidence and seawater intrusion once reused water is produced. Across the world, reused water has been distributed in several places such as Spanish coastal cities^4,82^, Italian and Greece coastal cities^83^, and Singapore^84^.

According to Giampietro et al.^62^ nexus is associated with dependent elements in the real world. In general, discussing one nexus element is relatively impossible as a nexus element is dynamically dependent on other elements. The CLD shows the dependence between nexus elements.

Growing coastal areas are associated with land-use change (land as a nexus element) and farming areas (food as a nexus element). Growing coastal areas require more water (a nexus element). This means that there are interlinked elements between water, food, and land. Once land-use change rises, more water is needed to support growing coastal areas (growing population and growing industries). Land-use change may deplete farming land which means that land-use change as a nexus element influences food supply as a nexus element.

The nexus concept also enables us to find sustainable solutions. As explained earlier, after the dike construction, people have still faced difficulties due to tidal floods. The dikes may hamper tidal floods, but excessive groundwater exploitation negates the benefit of the dikes, as the dikes have experienced land subsidence.

### Implications for other coastal areas

Table 1 highlights similar problems in Pekalongan and coastal cities around the world. Land-use change, floods (either flash floods or tidal floods), and water scarcities have occurred in other coastal areas. For instance, as Pekalongan, other coastal cities such as Mumbai and Chinese coastal cities (Table 1) have also experienced land use. Several coastal cities have also experienced other natural disasters such as tidal floods and water scarcity (Table 1).

**Table 1:** Similarities between Pekalongan and other coastal cities.

No.	Descriptions	Other coastal cities	Pekalongan
1	Land-use change due to urban settlements	Coastal cities such as Mumbai^85^, Bangladesh^86^, Alicante coastal cities^76^, and Chinese coastal cities^69^ have experienced land-use change due to population growth	Pekalongan has experienced land-use change due to population and economic growth^87^
2	Seal-level rise and inundated areas after tidal floods	Especially low-lying coastal cities may be inundated such as California^88^, Bangladesh^1^, Mumbai^85^, and Chinese coastal cities^69^	Experiencing inundated areas after tidal floods^47^
3	Water scarcity	Coastal cities such as Alicante coastal cities^76^, Japanese cities^16^, and coastal cities in USA^89^ have experienced water scarcity due to the gap between water demand (after economic growth and population growth) and water supply	Experiencing water scarcity as this city does not have sufficient water sources^87^
4	Flash floods and inundated areas after flash floods	Some coastal cities have experienced flash floods such as Mediterranean coastal cities^90^, Semarang^79^, Bangladesh^1^, Mumbai^85^, and Bangkok^91^	Besides tidal floods, Pekalongan has experienced flash floods due to excessed river supply^92^
5	Economic slowdown	Due to floods or water scarcity, coastal cities have experienced an economic slowdown as businesses lack water to provide proper services for their customers. For instance in Alicante coastal cities^76^ and Indian coastal cities^93^	Some companies cannot run their daily business due to inundated areas. Inundated areas also hamper industrial transportation and distribution^71^
6	Migration	Some coastal cities such as Bangladesh^1^, and Semarang (Indonesia)^79^ have experienced people migration due to inundated areas	People have left Pekalongan to find a comfortable space to live^71^

As the system archetypes are universal or generic patterns, identified structures of the system archetypes and proposed solutions described in this study can be founded in other coastal regions. Due to generic solutions of the system archetypes, suggested solutions such as reused water application, controlled groundwater exploitation, and restriction of urbanization/land-use change are universal solutions for other coastal cities.

## Conclusion

This study discusses the dynamics of coastal areas, especially LECZ by highlighting interlinked nexus elements in the Pekalongan coastal area, Indonesia. The system archetype approach enables us to capture complex connections and links between nexus elements such as water, land, food, and health. This study is probably the first study capturing the connection between health, people migration, and renowned nexus elements such as water and food. This is important as climate change and environmental changes can affect human health and people migration. Furthermore, through identified system archetypes, the dynamics of interlinked elements are explained as well as problems and possible solutions to achieve a sustainable coastal city.

This study also shows us that the Limits to Growth structure is dominant in LECZ as there are nine balancing loops and five reinforcing loops. This means that the growth engines’ so-called reinforcing loops such as economic growth, land-use change, and groundwater exploitation are hampered by water availability and land availability. Once the growth engines are not properly controlled, some natural hazards such as tidal floods and water scarcity may occur.

Although this study only discusses a single LECZ, the Pekalongan coastal areas, this study can be a compass to understand the dynamics of interlinked elements in LECZs. Table 1 shows similar patterns between Pekalongan and other coastal cities across the world. It is thus this study offers useful findings for other LECZs.

This study also proves that the system archetypes are a promising qualitative tool in the nexus arena. The system archetypes, as seen in this study, can provide us insights such as growth engines (reinforcing loops) and tradeoffs (the Limits to Growth structures) found in the nexus studies. In turn, this study complements existing studies^16^ that use quantitative approaches in nexus studies. In addition, because developing countries such as Indonesia usually have limited research data and limited research funding, the system archetypes are a suitable tool in investigating the nexus elements.

Following this study, the next avenue is to use and expand the free SD tool from another study^94^ to quantitatively assess the nexus of water, land, and food under a changing climate in coastal cities. It is hoped that the next avenue will be investigating the impacts of climate change and water scarcity in critical LECZ such as the Pekalongan coastal area.
